# The Strategies for Treating “Alzheimer’s Disease”: Insulin Signaling May Be a Feasible Target

**DOI:** 10.3390/cimb44120421

**Published:** 2022-12-07

**Authors:** Guanying You, Jinyi Yao, Qiong Liu, Nan Li

**Affiliations:** 1 Shenzhen Key Laboratory of Marine Biotechnology and Ecology, College of Life Sciences and Oceanography, Shenzhen University, Shenzhen 518055, China; 2 Shenzhen Bay Laboratory, Shenzhen 518055, China

**Keywords:** Alzheimer’s disease, insulin resistance, amyloid-beta, tau phosphorylation, microglia

## Abstract

Alzheimer’s disease (AD) is a neurodegenerative disorder characterized by senile plaques formed by amyloid-beta (Aβ) extracellularly and neurofibrillary tangles (NFTs) formed by hyperphosphorylated tau protein intracellularly. Apart from these two features, insulin deficiency and insulin resistance have also been observed in AD brains. Thus, AD has also been referred to as type 3 diabetes by some of the scientists in this field. Insulin plays a pivotal role in learning and memory and is involved in regulating tau phosphorylation though the PI3KAkt-GSK3b signaling pathway. Interestingly, recent studies revealed that in AD brains the microglia transformed into a disease-associated microglia (DAM) status in a TREM2-dependent manner to restrain the toxicity of Aβ and propagation of tau. This also correlated with PI3K-Akt signaling through the adaptor of TREM2. Whether insulin has any effect on microglia activation in AD pathology is unclear so far. However, many studies demonstrated that diabetes increased the risk of AD. In this review, we summarize the main strategies for curing AD, including lowering the level of Aβ, suppressing the phosphorylation of tau, the ablation and/or repopulation of microglia, and especially the supply of insulin. We also propose that attention should be given to the influences of insulin on microglia in AD.

## 1. Introduction

Alzheimer’s disease (AD) is known as the most common form of dementia that occurs with aging. The histopathological characteristics of AD are defined by extracellular deposits of amyloid-beta (Aβ) and intracellular neurofibrillary tangles (NFTs) formed by hyperphosphorylated tau protein. Apparently, the initiation of AD is closely associated with the extent of Aβ production, as evidenced by familial AD cases. Those who carry the mutated amyloid precursor protein (APP) and/or presenilin-1/2 (PS1/2, the components of γ-secretase) tend to acquire an accumulation of Aβ plaques in the brain and probably suffer AD earlier. However, the degree of dementia is more strongly correlated with NFT burden than Aβ senile plaques [1], raising the notion that AD may be a secondary tau pathology.

In 2018, a research framework was suggested by the National Institute on Aging and Alzheimer’s disease Association (NIA-AA) to unify the pathological definition and staging of AD based on biological construct [2]. Those biomarkers are grouped into Aβ (A), pathologic tau (T), and neurodegeneration (N). Given that 30–40% of cognitively unimpaired elderly persons have abnormal amyloid biomarkers at autopsy, this proportion of amyloid-positive individuals will perfectly match the number of diagnosed AD patients 15–20 years later. This framework advised that Aβ alone with a normal pathologic tau biomarker (A+T−) can be assigned the label “Alzheimer’s pathologic change”, which refers to the earlier phases of the “Alzheimer’s continuum”. However, it may not be sufficient to cause tauopathy and neurodegeneration that finally lead to cognitive disorder. In addition, when the biomarker of both Aβ and tauopathy are present (A+T+), the term “Alzheimer’s disease” can be used to delegate the later phases of the “Alzheimer’s continuum”. 

**Figure 1:**
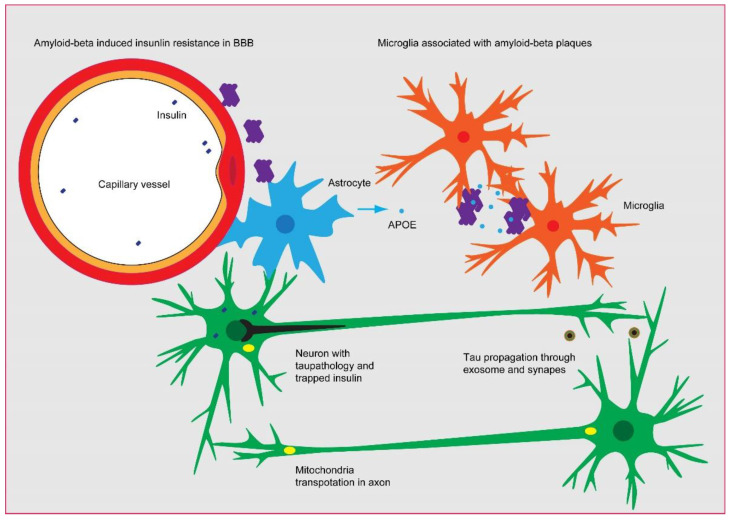
**The probable linkage between Aβ, insulin signaling, tau pathology, and microglia activation in AD.** The constant production of Aβ needs to be eliminated by microglia through endocytosis and/or by the drainage of micro-vessels, as evidenced by the association of Aβ plaques with microglia and micro-vessels in AD brain. However, the overloading of Aβ leads to the impairment of insulin sensing in the brain–blood barrier and parenchyma, which triggers the phosphorylation of tau and subsequently perturbs mitochondria and insulin secretion. The tau pathology could be propagated via the synapse and exosome in a microglia-dependent manner, eventually leading to neural atrophy.

However, the overloading of Aβ is speculated to be a causal factor for AD onset in the “Aβ cascade” hypothesis [3]. The subsequent tauopathy and neurodegeneration is considered to be more correlated with dementia and other clinical features of AD. Therefore, in considering the “Alzheimer’s continuum”, different therapeutic strategies have been adopted in AD treatment. For example, preventing the overproduction of Aβ and/or accelerating the clearance of Aβ, avoiding the hyperphosphorylation of tau, restraining the spread of tau, and arresting the activation microglia. Here, we review the relationship between Aβ accumulation, the insulin signaling pathway, tau hyperphosphorylation, and microglia activation (Figure 1), and we summarize different strategies for AD treatment, particularly the intermediary role of insulin signaling in AD pathology.

## 2. Restricting the Overload of Aβ

APP is a type I transmembrane protein, which is involved in regulating synaptic functions [4] and iron export [5]. APP has three common alternative splicing variants in the brain, and the size of each is 695, 751, and 770 amino acids, respectively. APP695 lacks the Kunitz-type protease inhibitor sequence in its ectodomain and is one of the most abundant proteins that is expressed by neurons. APP751 and APP770 are mainly expressed in glial cells. The cleavage of APP by α-secretase is a non-amyloidogenic pathway which produces a soluble APPα fragment and an 83-amino-acid C-terminal fragment (CTF-83). The CTF83 is further decomposed by γ-secretase, releasing a small P3 fragment into the extracellular space and the APP intracellular domain (AICD) into the cytoplasm. In contrast, the cleavage of APP by β-secretase is an amyloidogenic process, which releases a soluble APPβ ectodomain and a 99-amino-acid C-terminal fragment (CTF99). The cleavage of CTF99 by γ-secretase generates an Aβ and AICD fragment as well [6]. 

**Figure 2:**
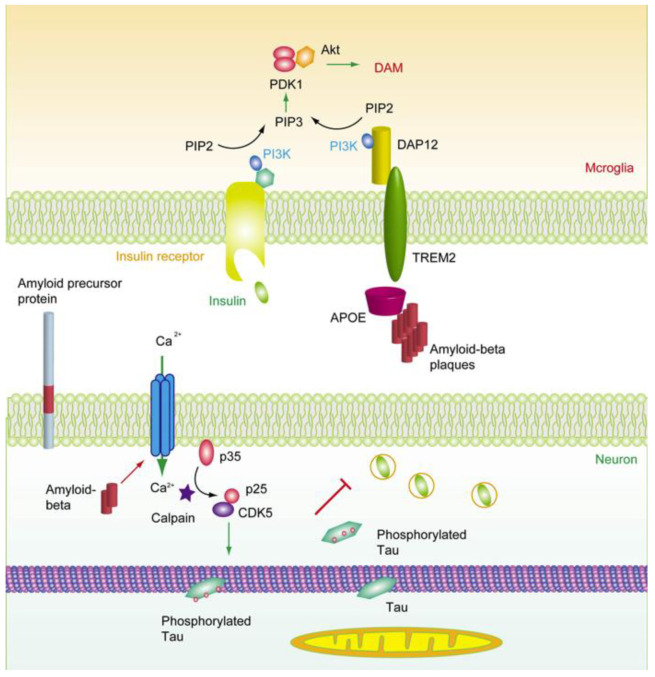
**The deductive role of insulin signaling in AD pathology.** Aβ induced calcium influx and the activation of calpain, which further triggered the activation of CDK5 through the cleavage of p35 [31]. CDK5 induced phosphorylation of tau, triggered the detachment of tau from microtubules, and in turn perturbed the function of mitochondria. On the other hand, the microglia switch homeostasis to DAM status in an APOE- and TREM2-dependent manner to protect neurons from the damaging effect of Aβ overproduction [32,33]. TREM2 functions via its adaptor DAP12 (DNAX activation protein of 12 kDa) and PI3K-Akt pathway, which is also regulated by insulin signaling. Thus, insulin can inhibit tau phosphorylation by suppressing GSK3β in neurons and help microglia to maintain the proper DAM status via PI3K-Akt pathway.

Mutations in the APP, ADAM10 (a disintegrin and metalloproteinase domain 10), and PS1/2 genes are closely related to the onset of AD. The APP gene is located on chromosome 21; not only do the mutations within and immediately flanking the Aβ region of APP cause an aggressive form of FAD, but an individual with trisomy 21 (Down’s syndrome) harboring three copies of APP also exhibits abundant diffuse Aβ plaques in their brain and invariably develops neuropathologically typical AD. In addition, mutations that attenuate secretase activity of ADAM10, the main α-secretase accounting for APP proteolysis, are associated with elevated Aβ levels [7,8]. Moreover, PS1 and PS2 are critical components of the γ-secretase complex. Missense mutations in PS1/PS2 are found to result in an increasing production of Aβ-42/43 peptides, which are an aggregation-prone species and lead to profound Aβ deposition [9]. The toxicity of soluble Aβ oligomer has been observed in various cellular processes. For example, Aβ oligomers could directly interact with membranes to form pores for ions and disrupt the proper permeability of the membranes [10], leading to the depolarization of neurons and microglia [11]. The soluble oligomers could also inhibit LTP through excessive activating of NR2B containing the NMDA receptor [12] (Figure 2) and perturb the synaptic plasticity through mitochondria [13,14]. In addition, Aβ oligomers could induce inflammation through receptor-mediated mechanisms [15] and impair the integrity of the blood–brain barrier [16]. 

Many efforts have been made to reduce the production of Aβ for the purpose of curing AD. For example, synthetic retinoid acitretin, which enhances the expression of ADAM10, the most effective α-secretase for APP, showed beneficial effects in AD patients [17]. In addition, an ADAM10 endocytosis inhibitor has been developed recently, which can upregulate the postsynaptic localization and activity of ADAM10 to increase the non-amyloidogenic process of APP [18]. On the other hand, the inhibitor of the β-site amyloid precursor protein cleaving enzyme-1 (BACE1), namely, β-secretase, has also been tested in the clinic. For example, verubecestat and lanabecestat could reduce the level of Aβ in cerebrospinal fluid by 63% to 81%. However, this had no beneficial effect on cognition, other than adverse side effects including sleep disturbance, weight loss, and decreased appetite [19,20]. Moreover, small molecule inhibitors of γ-secretase, such as semagacestat and avagacestat, have been found to successfully reduce Aβ production in AD transgenic mice and patients. Nevertheless, due to the nonselective inhibitory effects on both APP and Notch, they failed in clinical trials [21].

In the brain, ADAM10 is mainly localized in the synapse. Knockout of ADAM10 results in embryonic death in E9. BACE1 is usually found in the plasma membrane in the endosome and Golgi apparatus, functioning at an optimal pH of 4.5. Knockout of BACE1 leads to diabetes and hypomyelination. In contrast to ADAM10 and BACE1, γ-secretase is a transmembrane protein complex containing presenilin, nicastrin, anterior pharynx defective 1 (Aph-1), and presenilin enhancer-2 (Pen-2). The catalytic site of γ-secretase is located in the PS subunit, which has two homologs in mammalian cells, PS1 and PS2. Knockout of PS1 results in Notch signaling deficiency and is lethal for mice. PS2 knockout mice are normal. Moreover, both nicastrin and Aph-1 knockout mice have shown embryonic lethality. ADAM10, BACE1, and γ-secretase have many other substrates apart from APP, including the components of the Notch signaling pathway and other transmembrane proteins such as Neuroligin 1 and Neuregulin. BACE1 also plays an important role in insulin signaling conduction by the cleavage of the insulin receptor, reducing its expression on the cell surface [22,23]. Therefore, it is not surprising that the inhibition of BACE1 and γ-secretase result in undesirable outcomes. 

Another way of lowering the accumulation of Aβ is to accelerate its clearance in the brain, either through the phagocytosis of microglia or the drainage of micro-vessels. It has been clearly demonstrated that the dysfunction of Aβ clearance is associated with the late onset of AD. For example, the rate of transport across the blood–brain barrier and perivascular drainage to the systemic circulation was slowed down for the Aβ Dutch variant compared with Aβ WT. Additionally, the APOE4 variant—the strongest genetic risk factor for AD except for mutations in APP and PS1/2—could affect the endocytosis process and increase Aβ accumulation at the blood–brain barrier (BBB) [24]. In addition, the cell surface triggering receptor expressed on myeloid cells 2 (TREM2), which is expressed in microglial, is found to be upregulated by Aβ and facilitates the phagocytosis of Aβ. The R47H mutation in TREM2, which is also one of the strongest genetic risk factors for AD, perturbed the activation of microglia and led to Aβ deposition [25]. Moreover, phospholipase D3 (PLD3), bridging integrator 1 (BIN1), phosphatidylinositol-binding clathrin assembly protein (PICALM) [26], and sortilin-related receptor (SORL1)—the next strongest genetic risk factors of AD after APOE4 and TREM2—were also involved in regulating the endocytosis process [27,28]. This indicates that the clearance of Aβ is an important way of curing AD. To improve the acceleration of Aβ, the monoclonal antibody of Aβ oligomers, such as aducanumab, has been clinically used [29]. It significantly reduced the level of Aβ plaque accumulation. However, the side effects, such as encephaledema, were also observed in a large portion of the subjects [30], implying that the elimination of Aβ through antibody-mediated endocytosis is feasible in AD, but researchers should be cautious of overactivating microglia and impairing endothelial cells in BBB.

## 3. Rescuing Insulin Signaling

In 2005, Eric et al., proposed to use “Type 3 diabetes” to reflect the abnormal changes in the levels of insulin, insulin-like growth factor (IGF) I/II, insulin receptor (IR), and insulin receptor substrate (IRS) in the brains of AD patients [34]. IR and IGF receptors were found to be highly expressed in neuron and glial cells throughout the brain [35,36]. However, more recently, single-cell transcriptomic analyses have indicated that the mRNA transcript of IR is found in higher concentrations in endothelial cells in the brain [37]. The insulin-induced activation of IR at the BBB was blunted in transgenic AD model mice [38,39]. Meanwhile, insulin deficiency was observed in mild cognitive impairment patients and early-stage AD patients [40]. Importantly, it has also been demonstrated that Aβ has a similar tertiary structure to insulin, leading to Aβ being a competitive inhibitor for insulin. Aβ oligomers have been shown to inactivate IRS-1 and reduce its level [41], which in turn abolishes the inhibitory activation of insulin on glycogen synthase kinase -3 (GSK-3β) and further results in tau hyperphosphorylation [42].

Insulin comes from either synthesis de novo in the brain [43] or passing through the blood–brain barrier (BBB) from the plasma to the brain via the capillary endothelial cells by a selective, saturable, receptor-dependent mechanism [44,45]. By binding to the IR and IGF receptors, insulin facilitates the phosphorylation of IRS and subsequently activates phosphatidylinositol-3-kinase (PI3K) and AKT (protein kinase B, PKB). Thus, the glucose transporter 4 (GLUT4) in cytosol is recruited onto the plasma membrane to promote glucose uptake [46]. Insulin-induced transportation of GLUT4 plays an important role in hippocampal-dependent memory [47]. The activation of AKT further regulates the activity of the mammalian target of rapamycin (mTOR), GSK-3β, and cAMP-responsive element-binding protein (CREB). The mTOR pathway regulates various cellular functions, including glucose metabolism, mitochondrial oxidative respiration, and autophagy. Of note, GSK-3β is involved in the phosphorylation of tau as well as energy metabolism. On the other hand, IRS can also activate growth factor receptor-bound protein 2 (Grb2) which in turn stimulates SOS1 and Ras, Raf, and mitogen-activated protein kinases (MAPK) [48]. Interestingly, APOE is also involved in modulating PI3K/Akt signaling [49,50]. The APOE4 variant can reduce the levels of insulin receptor substrate-1 and PI3K, decrease Akt phosphorylation [51], and impair neuronal insulin signaling by trapping the insulin receptor in the endosomes [52]. Moreover, the activation of TREM2 also modulated PI3K/Akt signaling [53,54]. The mutation of TREM2, which is correlated with the onset of AD, impaired its activity on PI3K-AKT-GSK3β through SYK [55]. The activation of microglia mediated by TREM2 also regulated the phosphorylation of tau through GSK3β [56] (Figure 2).

AD patients showed lower CSF insulin levels, higher plasma insulin levels, and reduced CSF/plasma insulin ratios compared to healthy controls [57]. It has been reported that the insulin-degrading enzyme (IDE) level was reduced in the hippocampus of AD patients; however, it was increased in the micro-vessels in AD with CAA [58]. IDE is able to degrade both insulin and Aβ [59] as well as many other molecules with amyloidogenic potential, such as glucagon, amylin, calcitonin, and atrial natriuretic peptide [58]. It has also been observed that in mice lacking IDE the accumulation of endogenous brain soluble Aβ was increased. In contrast, transgenic overexpression of IDE in neurons reduced the brain soluble Aβ level and delayed amyloid plaque formation in APP transgenic mice. In addition, the level of IDE in theAPOE4 carrier was reduced by approximately 50% [60], indicating that the shortage of insulin in AD brain may enhance Aβ accumulation through downregulating the IDE level. On the other hand, in the insulin knockout mice model, the phosphorylation of tau was enhanced, thereby leading to the formation of NFTs [61]. Using streptozotocin (STZ) to deplete insulin could also induce the tau pathology [62]. Insulin receptor substrate 2 (IRS2) knockout could promote the phosphorylation of tau as well [63]. Collectively, this evidence consistently demonstrates that insulin signaling dysfunction and abnormal insulin levels have a profound influence on AD pathology. 

A previous study indicated that intranasal insulin administration was able to improve memory in humans [64]. The verbal memory in AD and MCI subjects without the APOE4 allele was improved 15 min after intranasal insulin 40 IU administration, and the plasma insulin or glucose levels were not perturbed [65]. Another trial administrated a placebo or 20 IU or 40 IU of insulin detemir with a nasal drug delivery device to treat adults diagnosed with MCI or mild to moderate AD. A 21-day treatment with 20 IU of insulin increased the plasma concentration of the Aβ-40 without affecting the level of Aβ-42, resulting in an increased Aβ 40/42 ratio [66]. The results also showed that 40 IU significantly improved the verbal working memory and visuospatial working memory. However, these effects were modulated by the APOE status. Insulin treatment reduced the insulin resistance in APOE4 carriers but not in APOE4-negative ones [67]. Craft et al., also reported that regular insulin treatment reduced the tau-p181/Aβ42 ratio in plasma and preserved the brain volume [68]. The intranasal administration allowed insulin arriving in the CNS to bypass the periphery and prevents the risks associated with hypoglycemia [69]. However, more recently, in a randomized clinical trial of 289 adults with mild cognitive impairment or AD, the intranasal insulin treatment showed no cognitive or functional benefits for the patients [70], but the limitation in this study was that the device used for intranasal insulin supply had not been tested before. Thus, further studies are still required to ascertain the underling mechanism of how insulin supply may work for curing AD.

Insulin sensitizers that were used in treating type 2 diabetes also showed positive effects for curing AD. The peroxisome proliferator-activated receptor (PPAR-γ) agonists such as pioglitazone [71] and rosiglitazone [72] improved memory and stabilized plasma Aβ42 concentrations. The administration of 10–30 mg/day of pioglitazone for 6 months to AD patients accompanied with type 2 diabetes mellitus decreased fasting plasma insulin levels. The administration of rosiglitazone 4 mg/day for a 6-month course improved the delayed recall and selective attention of AD subjects. The plasma Aβ levels were not increased with the progression of AD and declined compared with the control group which received a placebo [72,73]. Another study accomplished by Risner et al., demonstrated that 8 mg/day of rosiglitazone treatment for 24 weeks significantly improved the non-APOE4-positive AD patients [74]. However, a phase 3 trial showed no effects on cognition, regardless of APOE type [75]. The effects of antidiabetic drugs are also under evaluation in AD therapy. In transgenic AD mice, metformin increased the IDE level [76] and prevented amyloid plaque deposition and memory impairment [77]. A study on primary neurons from wild-type mice showed that metformin induced the PP2A-dependent dephosphorylation of tau [78]. Clinically, the use of metformin showed protective effects on brain volumes in non-demented elderly individuals with diabetes [79]. For mild cognitive impairment or mild dementia due to AD, metformin also improved executive functioning [80]. The vanadium compounds that were used in treating diabetes also showed a protective effect in AD transgenic mice models through regulating PPARγ [81,82,83]. However, it is unknown whether these antidiabetic agents are efficient in the late stage of AD.

## 4. Preventing Tau Pathology

Although Aβ overload is believed to be the most important risk factor for AD development, it is worth noting that there are a great many people bearing Aβ plaques in their brains who do not exhibit dementia symptoms unless the tau pathology or cerebral amyloid angiopathy (CAA) occurs. On the other hand, it was demonstrated that the presence of Aβ plaques facilitated local tau seeding in dystrophic neurites that led to the spreading and formation of phosphorylated forms of tau in neuritic plaques and NFTs in mice [84]. In addition, the reduction in tau has been found to protect neurons from the loss of mitochondrial membrane potential [85], excitotoxicity [86], and axonal transport inhibition [87] induced by Aβ [88], indicating that tau is a critical target for AD treatment. Tau is a microtubule-associated protein involved in microtubule stabilization and intracellular cargo transport. Tau is encoded by the *MAPT* gene, which is located on chromosome 17. In the human brain, exons 2 and 3 of *MAPT* account for the two N-terminal repeats (N), while exon 10 encodes the second microtubule-binding repeat (R) of four in total. Therefore, the alternative splicing of *MAPT* yields six distinct isoforms of tau, which are 0N3R, 0N4R, 1N3R, 1N4R, 2N3R, and 2N4R. Knockout of tau led to glucose intolerance [89] and impaired the hippocampal response to insulin by modulating the phosphatase and tension homologue on chromosome 10 (PTEN) [90]. Tau deletion also contributed to the accumulation of iron in the brain, resulting in conditions such as Parkinson’s disease [91]. However, it has also been found that in a type 1 diabetes model induced by STZ, tau knockout attenuated the cognitive impairment triggered by insulin deficiency [92], whereas human tau transgenic mice showed robust deficits in learning and memory processes under the same conditions [93]. These observations suggest that tau itself is closely related to cell signaling implementation rather than only taking part in stabilizing microtubules. 

Notably, tau pathology is not only presented in AD but also associated with many other tau pathologies, such as chronic traumatic encephalopathy (CTE), a subclass of frontotemporal dementia with Parkinsonism linked to chromosome 17 (FTDP-17tau); Pick’s disease (PiD); progressive supranuclear palsy (PSP); corticobasal degeneration (CBD); and argyrophilic grain disease (AGD) [94]. As mentioned above, in AD the neurofibrillary tangles formed by tau aggregation usually initiate in the neurons of the medial temporal lobe regions (i.e., hippocampus, entorhinal cortex, and amygdala) in the form of both 3R and 4R tau. Likewise, in CTE, the tau filaments are also detected in frontal and temporal cortices in the form of both 3R and 4R. However, in PiD, tau pathology is mainly found in granular neurons in the hippocampal dentate gyrus, the hippocampal CA1 pyramidal neurons, and layer II of the frontal and temporal cortices in the form of 3R, while in PSP, CBD, and AGD it mainly presents in the form of 4R in astrocytes. The affected regions include the basal ganglia, subthalamic nucleus, substantia nigra, and limbic lobe. 

Mutated human tau has been found in familial primary tau pathology but not in AD. Many mutations function in reducing the affinity of tau to microtubules, facilitating its phosphorylation by altering the interaction with other proteins, impairing the splicing of exon 10, or promoting the aggregation of tau into a beta-sheet structure. The mutation of tau is also known to induce insulin resistance [95,96] by increasing pSer-IRS1 [97] and to cause insulin accumulation as oligomers [98]. In addition, the *MAPT* gene has two main haplotypes, namely, H1 and H2, as a result of a 900 kb inversion in the q21 region of chromosome 17. The H1/H1genotype is considered a risk factor for PSP, CBD, and AGD. Moreover, the H1/H2 genotype confers a greater risk of developing dementia before the age of 45 years in individuals with Down’s syndrome. Tau species are capable of propagating from neuron to neuron through exosomes [99]. The propagation of tau raised the notion that tau is a prion protein which spreads in a conformational, strain-specific manner [100], and the toxicity of tau spreading is dependent on endogenous tau [101]. These above observations suggest that AD is likely a secondary tau pathology.

In the NFTs of AD and other tau pathologies, tau is hyperphosphorylated. The hyperphosphorylation of tau results in its dissociation from the microtubules; the detached tau misfolds and begins to aggregate and form the NFT. The posttranslational modifications of tau play an important role in tau aggregation. The phosphate group, methyl groups, and acetyl group to lysine residue change the basic character of tau. To evaluate the effects of phosphorylation on aggregation, scientists used Asp and/or Glu to replace the Ser320 and Ser324 on the R3 fragment of tau, which is the core of the NFT, and found that these pseudophosphorylations enhanced the aggregation of tau [102,103]. In another study, Briner et al., showed that in Src family non-receptor tyrosine kinase Fyn knockout mice the tau hyperphosphorylation was reduced, along with the near-complete ablation of NFTs [104]. However, when using chemical methods to synthesize the phosphorylated K18 fragment (four microtubule-binding repeats), the study showed that the phosphorylation at S258, S262, and S356 dramatically reduced the aggregation and fibril seeding activity of K18 in vitro [105]. Similarly, the phosphorylation at Tyr310 were also found to inhibit the aggregation and microtubule-binding activity of full-length tau and the K18 fragment [106]. Overall, it seems that the phosphorylation at different residues may have an alternative influence on the aggregating property [107]. Additionally, the phosphorylation of tau at the KXGS motifs in the C-terminal microtubule-binding domains prevented the ubiquitination and degradation of tau by proteasome. Likewise, the phosphorylation at S293, S324, and S356 inside the microtubule-binding domains diminished its degradation through the lysosome system. However, it seems that the NFT itself is not the direct reason for cognitive decline or neuronal death [108], whereas the phosphorylated tau promoted mitochondrial dysfunction in neurons with an FTLD mutant by impairing complex I of the electron transport chain [109,110]. The phosphorylated tau has also been demonstrated to interact with dynamin-related GTPase Drp1, thus promoting mitochondrial fission and morphology change [111]. Besides the hyperphosphorylation, the cleavage of tau has been found to precede and promote the tangle formation [112]. More importantly, the cleaved tau also induced the dysfunction of mitochondrial dynamics [113,114] (Figure 2).

Multiple kinases are involved in tau phosphorylation, including GSK-3β [115], AMP-activated protein kinase (AMPK) [116], cyclin-dependent protein kinase 5 (CDK5), CDK2, and CaMKII. Conversely, the dephosphorylation of tau is mainly afforded by protein phosphatase 2A (PP2A) [117]. Since the abnormal phosphorylation of tau is closely correlated with the aggregation, accumulation, and toxicity of tau pathology, the molecules that inhibit tau phosphorylation have been tested in curing AD. The functions of GSK3β have been extensively studied in cell proliferation, embryonic development, and immune response. It has been well established that phosphorylation at serine 9 and 389 inhibits GSK3β activity whereas phosphorylation at tyrosine 216 increases its activity. Lithium is a selective GSK3β inhibitor which acts by competition with magnesium in the ATP-binding pocket. In vitro and in vivo studies clearly showed that lithium treatment effectively reduced tau phosphorylation [118]. Other non-ATP-competitive GSK3β inhibitors, such as Tideglusib [119,120], oxadiazole containing small molecules, and pyrimidinone containing small molecules also showed beneficial effects in an AD model [121]. However, the adverse effects of GSK3β inhibitors were monitored in control animals [120], suggesting that a more specific inhibitor of GSKβ on tau phosphorylation is needed in future studies. In contrast to the kinases that induce the phosphorylation of tau, PP2A is responsible for the dephosphorylation of tau. In the brain of AD patients, the protein level and phosphatase activity of PP2A was reduced [122]. Transgenic mice with reduced PP2A activity presented somato-dendritic accumulation of hyperphosphorylated and aggregated tau in cortical pyramidal cells [123]. Using okadaic acid to inhibit PP2A and PP1 also increased tau phosphorylation [124]. The sphingosine-1-phosphate receptor selective agonist SEW2871 has been shown to reduce the tau Ser262 phosphorylation via the AMPK-PP2A pathway [125]. In addition, the zinc chelator clioquinol has been demonstrated to be capable of elevating PP2A activity and deceasing PP2A Tyr307 phosphorylation [126]. However, the problem with these PP2A agonists remains because of lacking specificity for different substrates. 

## 5. Microglia Depletion and Repopulation

Microglia are innate immune cells in the brain which originate from erythromyeloid progenitor cells in the yolk sac [127]. Microglia exhibit a remarkable capacity for proliferation and self-regeneration in the central nervous system [128]. In the brain, microglia serve as resident phagocytes, playing an important role in pathogen defense and injury response. Microglia are also involved in sculpting synapses by phagocytizing inappropriate synaptic connections, which is necessary for normal brain development [129]. In the brain of AD patients, microglia are stably associated with Aβ deposition [130], and they are responsible for Aβ uptake and clearance [131]. However, Aβ aggregates can act as disease-associated molecular patterns and trigger microglia activation through pattern recognition receptors, such as Toll-like receptors and NRLP3 inflammasomes [132,133], leading to the secretion of TNFα, IL1β, and other inflammatory cytokines. Single-cell sequencing revealed that the mRNA profiles of microglia in the brain of AD patients were switched from the homeostatic stage to the disease-associated microglial (DAM) stage [134,135].

Chemically or genetically activating microglia significantly accelerated tau pathology and behavioral abnormalities in the human tau mouse model of tauopathy [136,137]. In addition, deleting the microglia protein Cx3cr1 in transgenic tau models showed that the onset and progression of tau pathology were accelerated by artificially activating microglia. Moreover, APOE4 variants were found to exacerbate the tau pathology in tau P301S transgenic mice in a microglia-dependent manner [138]. However, other studies demonstrated that the activated microglia mitigate Aβ-associated tau seeding and spreading [139]. TREM2 deficiency and mutation leads to a decrease in Aβ-plaque-associated microglia and facilitates the seeding and spreading of neuritic plaque tau aggregates [140,141]. It was suggested that, although DAM attenuated the progression of neurodegeneration in certain mouse models, inappropriate DAM activation accelerates neurodegenerative disease [142]. Interestingly, APOE [49] and TREM2 [55] are both implicated in regulating the DAM status through the PI3K and Akt signaling pathways (Figure 2). It is also worth noting that the mutation of TREM2 is not only associated with the onset of sporadic AD but also correlated with Parkinson’s disease, ALS, and frontotemporal dementia [143]. 

Microglia are critically dependent on the colony-stimulating factor-1 receptor (CSF1R) for their survival [144]. CSF1R is expressed on all myeloid cells, so the signaling interference through this receptor will not only affect microglia cells but also influence peripheral macrophages [145]. For this reason, *Csf1r^−/−^* mice display mononuclear phagocyte deficiency, neurodevelopmental abnormality, and a shortened lifespan [146,147]. Thus, a pharmacological method to deplete microglia were used in the AD model. GW2580 is the first reported CSF1R-kinase inhibitor as well as the first CSF1R inhibitor utilized in a mouse model of AD [148,149]. It blocks microglial proliferation, shifts the microglial transcriptomic profile to an anti-inflammatory profile in APP/PS1 mice, and prevents cognitive decline, although it did not modify the burden of Aβ [149]. Although the microglia are capable of clearing Aβ, the depletion of microglia in mice with established brain amyloid had no effect on Aβ deposition but resulted in less spine and neuronal loss [150]. It is suggested that following the initial period of plaque formation, microglia surround the plaques and subsequently mount a harmful and non-resolving inflammatory response; however, prolonged depletion of microglia throughout the plaque-forming period impaired the plaque formation, compaction, and growth [151].

Pexidartinib is a selective CSF1R/KIT/FLT3 inhibitor [152]. Treatment with pexidartinib enabled the depletion of more than 99% of all microglia for 3 or 8 weeks in adult mice with no deficits in any behavioral cognitive task administered [153,154]. The depletion of microglia by a CSF1R inhibitor was found to arrest tau propagation in PS19 mice and in C57 mice that were forced to express tau by an adeno-associated virus (AAV) [155], suggesting that microglia are involved in the cell-to-cell spread of tau. In mice with forced P301L tau overexpression, Calton et al., found that the depletion of microglia using PLX5622 dramatically reduced the propagation of phosphorylated tau [156]. Casali et al., demonstrated that in 5xFAD mice PLX5622 elicited microglial repopulation and subsequent plaque remodeling, resulting in more compact plaques predominating microglia-repopulated regions. Microglia limit diffuse plaques by maintaining compact-like plaque properties, thereby blocking the progression of neuritic dystrophy [157]. Similarly, Gratuze et al., found that after microglia depletion using PLX3397, repopulated microglia clustered around plaques, and they found a reduction in disease-associated microglia (DAM) gene expression [139]. However, another study stated that the repopulation of microglia induced by PLX5622 did not impact the amyloid pathology in 3xAD model mice but did change the phosphorylation style of tau [158]. 

## 6. Conclusions and Perspectives

To date, many hypotheses of AD etiology have been proposed based on clinical research and experimental data, including the amyloid cascade hypothesis [159], cholinergic hypothesis [160], neuroinflammatory hypothesis [161], mitochondrial hypothesis [162], oxidative stress hypothesis [163], insulin resistance hypothesis [164], and calcium hypothesis [165]. They are also supported by substantial evidence. In this review, we tried to piece together the evidence and find the key nodes that link amyloid-beta overproduction, neuroinflammation, insulin resistance, tau pathology, mitochondrial impairment, and neuron death. 

Previously, the Aβ oligomer mono-antibody aducanumab had been approved by the FDA; it showed great efficiency in clearing Aβ from the parenchyma of the brain, though side effects, such as encephaledema, were also seen (Table 1). These observations suggest that accelerating the clearance of Aβ with an antibody is viable with the proper dose in an earlier stage of AD. However, with the presence of tau pathology it may not be enough to stop the progress of AD only by reducing the level of Aβ. Based on the evidence collected, we propose that the insulin signaling pathway may act as a linkage between Aβ, tau pathology, and microglia activation. Efficient insulin supply may prevent or postpone the initiation of tau pathology. On the other hand, it may be helpful to maintain microglia homeostasis through the PI3K-Akt pathway to restrain the propagation of tau (Figure 2). 

## Figures and Tables

**Table 1:** Overview of different strategies for the treatment of AD.

Strategies	Targets	Drugs or Methods	Anticipating Function	Side Effects
Lowering Aβ	ADAM 10	Retinoid acitretin PEP3	Activating ADAM10 Upregulating the postsynaptic localization and activity of ADAM10	Perturbed unspecific substrates besides APP
	BACE1	Verubecestat Lanabecestat	BACE1 inhibitor	Perturbed unspecific substrates besides APP
	γ-secretase	Semagacestat Avagacestat	γ-secretase inhibitor	Perturbed unspecific substrates besides APP
	Aβ-oligomer	Aducanumab	Aβ-oligomer antibody	Encephaledema
Increasing insulin signaling	Insulin deficiency	Intranasal insulin administration	Increasing insulin level in CNS	-
	Insulin insensitivity	Metformin	Increasing insulin insensitivity	-
	PPARγ	Pioglitazone Rosiglitazone	Increasing insulin insensitivity	Edema and weight gain
Limiting tau phosphorylation	GSK3β	Lithium Tideglusib	GSK3β inhibitor	Perturbed unspecific substrates besides tau
	PP2A	SEW2871 Zinc chelator clioquinol	PP2A activator	Perturbed unspecific substrates besides tau
Restrict inflammations	Microglia	GW2580 PLX5622 PLX3397	Depletion and repopulation of microglia	-
